# Rare occurrence of pseudomyxoma peritonei (PMP) syndrome arising from a malignant transformed ovarian primary mature cystic teratoma treated by cytoreductive surgery and HIPEC: a case report

**DOI:** 10.1186/s12957-022-02548-8

**Published:** 2022-03-11

**Authors:** Francesca Ponzini, Luke Kowal, Mariam Ghafoor, Allison Goldberg, Joanna Chan, Ryan Lamm, Shawnna M. Cannaday, Scott D. Richard, Avinoam Nevler, Harish Lavu, Wilbur B. Bowne, Norman G. Rosenblum

**Affiliations:** 1 Sidney Kimmel Medical College at Thomas Jefferson University, Philadelphia, PA USA; 2 Department of Pathology, Thomas Jefferson University Hospital, Philadelphia, PA USA; 3 Department of Surgery, Thomas Jefferson University Hospital, Philadelphia, PA USA; 4 Division of Gynecologic Oncology, Department of Obstetrics and Gynecology, Thomas Jefferson University Hospital, Philadelphia, PA USA

**Keywords:** Pseudomyxoma peritonei, Ovarian, Teratoma, Appendiceal mucocele, Disseminated peritoneal adenomucinosis, Hyperthermic intraperitoneal chemotherapy (HIPEC), Cytoreductive surgery (CRS)

## Abstract

**Background:**

Pseudomyxoma peritonei (PMP) syndrome is a disease process that typically occurs from ruptured appendiceal mucocele neoplasms. PMP syndrome arising from malignant transformation of an ovarian primary mature cystic teratoma (MCT) is a pathogenesis rarely encountered.

**Case Presentation:**

Herein, we report a 28-year-old patient evaluated and treated for a right ovarian mass and large volume symptomatic abdominopelvic mucinous ascites. Molecular profiling and genetic analysis revealed mutations in ATM, GNAS, and KRAS proteins while IHC demonstrated gastrointestinal-specific staining for CK20, CDX2, CK7, and SATB2. Peritoneal cytology showed paucicellular mucin. Diffuse peritoneal adenomucinosis (DPAM) variant of PMP arising from a ruptured ovarian primary MCT after malignant transformation to a low-grade appendiceal-like mucinous neoplasm was ultimately confirmed. Treatment included staged therapeutic tumor debulking and right salpingo-oophorectomy followed by cytoreductive surgery and hyperthermic intraperitoneal chemotherapy (HIPEC).

**Conclusions:**

Our report builds upon the existing literature supporting this aggressive treatment option reserved for advanced abdominal malignancies utilized in this patient with a rare clinical entity.

## Introduction

Pseudomyxoma peritonei (PMP) is a rare condition associated with ruptured mucinous neoplasms that typically originate from the appendix. PMP arising from malignant transformation of an ovarian teratoma is exceedingly rare with a reported incidence of 1-2/1,000,000 per year [1]. PMP syndrome presents with a variable spectrum of disease biology and malignant potential but commonly results in widespread mucinous ascites disseminated throughout the abdomen and pelvis [2, 3]. If left untreated, patient demise occurs from mass effect of large accumulations of mucinous ascites and tumor-causing bowel obstruction, perforation, and related sequela [4]. Computed tomography (CT), magnetic resonance imaging (MRI), and expert pathologic review are mandatory to establish PMP diagnosis prior to commencing with treatment [5].

Definitive treatment for PMP is complete gross resection and/or cytoreductive surgical (CRS) removal of all visible macroscopic tumor burden followed by hyperthermic intraperitoneal chemotherapy (HIPEC) [5, 6]. CRS and HIPEC is a complex surgical procedure that requires a multidisciplinary team approach [7]. At our institution, a specialized team of surgeons routinely provides this treatment in our appendix-derived PMP patients with optimal oncologic outcomes and low morbidity [8].

In patients with ovaries, determining the origin of PMP is challenging when a non-appendiceal source is suspected, implicating the possibility of a rare ovarian mucinous tumor etiology. Historically, Ronnett et al. classified PMP as being ovarian in origin if the appendix cannot be identified and/or if a rupture of a mucinous tumor of the appendix goes undetected [2].

Herein, we describe a very rare occurrence of PMP syndrome arising from a ruptured low-grade appendiceal-like mucinous neoplasm that evolved from a transformed ovarian primary mature cystic teratoma (MCT) in a young patient that was definitively treated by cytoreductive surgery and regional intraperitoneal perfusion with hyperthermic chemotherapy.

## Case report

**Fig. 1:**
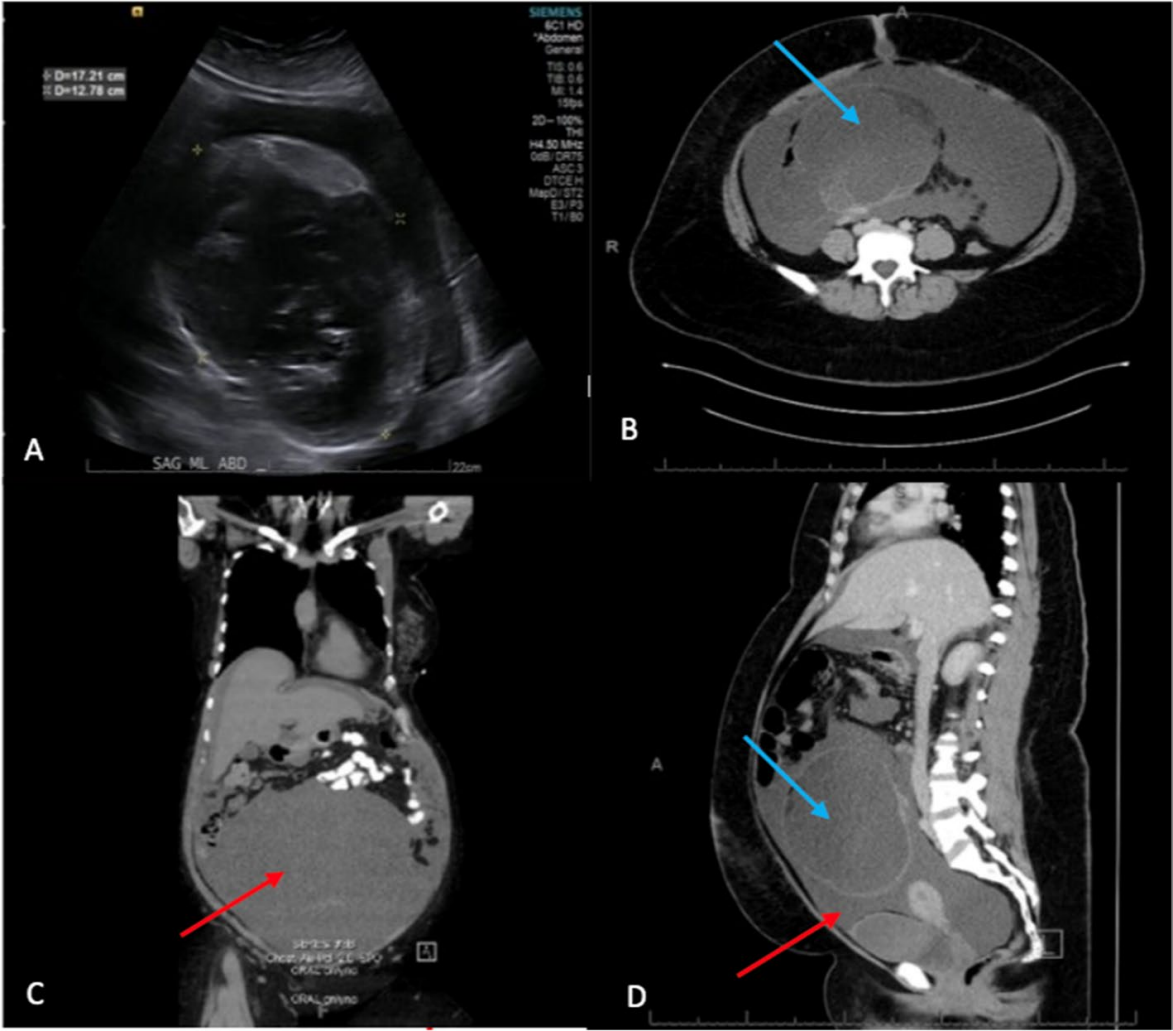
**A** Ultrasound images of the 18-cm mass. **B**–**D** Contrast-enhanced perioperative abdominal CT scan of the 18cm mass in the lower abdominal cavity (**B** axial, **C** coronal, **D** sagittal). The imaging revealed a moderately large volume of mucinous ascites (red arrows) and a large right ovarian mass (blue arrows) concerning for cystic teratoma vs malignancy

A 28-year-old female presented to the emergency department for a gynecological consultation after imaging performed at an outside hospital showed a large right ovarian mass concerning for malignancy. The patient’s presentation followed two months of upper abdominal pain aggravated by movement, coincident with weight loss, shortness of breath, abdominal distention and decreased appetite. Her ultrasound and CT scan showed an 18cm abdominopelvic mass originating from the right ovary which was cystic in appearance containing soft tissue, fatty and calcified components. Importantly, there was a large volume symptomatic mucinous appearing ascites (Fig. 1). Serologic tumor markers including CA-125, CA19-9, and CEA, were normal. Past medical, surgical, social, and family history were non-contributory.

**Fig. 2:**
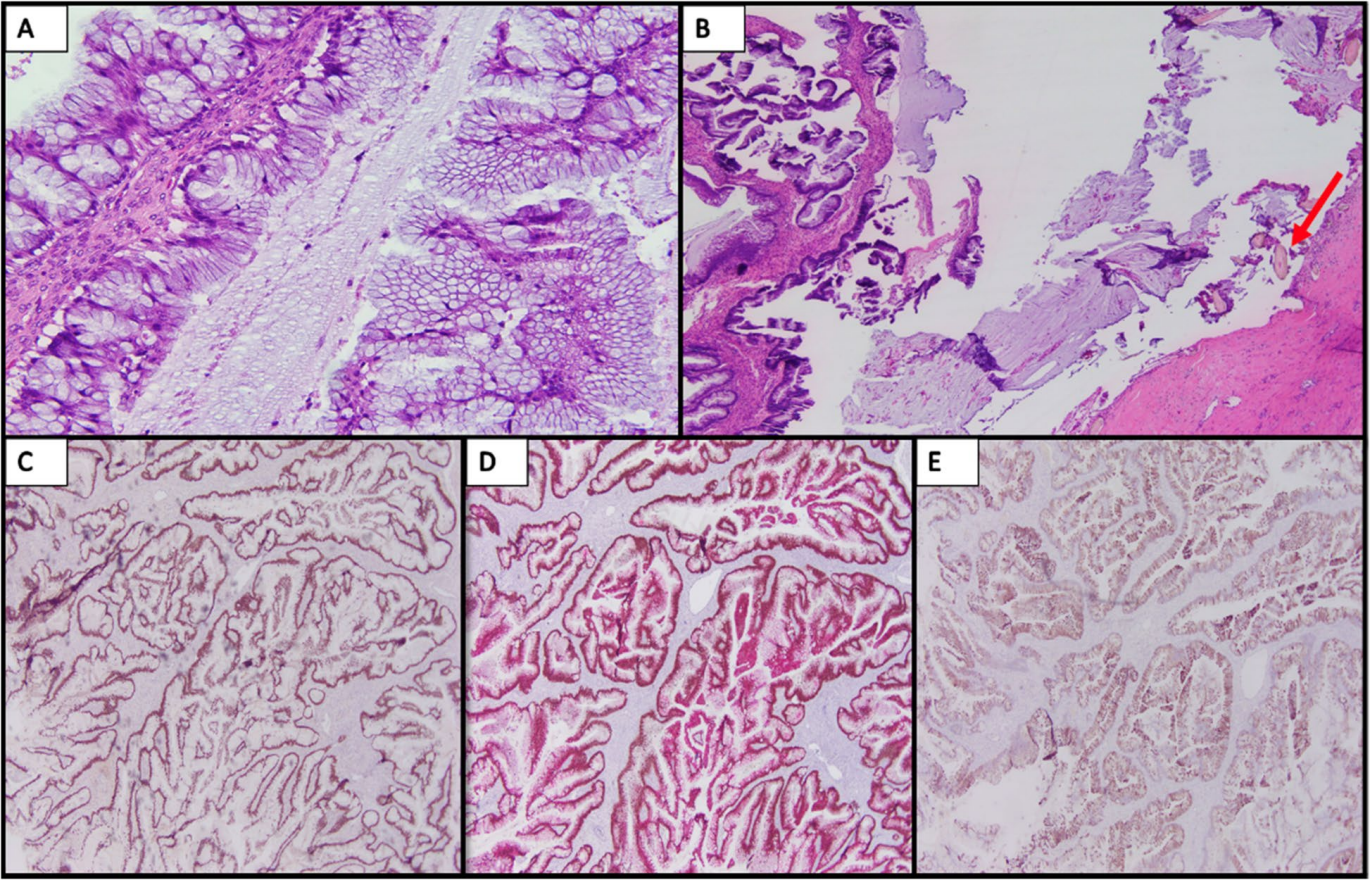
**A** Mucinous neoplasm, right ovary, showing complex mucinous epithelium (200×). **B** Hair follicles (red arrow) with adjacent mucinous neoplasm (40×). **C** SATB2 showing strong nuclear staining (40×). **D** CDX2/CK20 dual stain showing strong nuclear and cytoplasmic staining, respectively (40×). **E** CK7 showing focal cytoplasmic staining

The patient underwent a two-staged operative approach. The index procedure included debulking of symptomatic mucinous ascites and resection of a pelvic mass requiring a right salpingo-oophorectomy. An 18-cm right ovarian tumor was described within a large volume of mucinous ascites. Pathology review demonstrated a mature cystic teratoma containing a mucinous epithelial component (Fig. 2). The ovarian mass contained multiple thick mucoid fluid-filled cysts ranging in size from 3 to 15cm. In addition, the mass appeared grossly necrotic with soft tan-gray areas interspersed among follicles of mature hair. The fallopian tube was not involved. Neoplastic cells on immunohistochemistry (IHC) were strongly positive for CK20, CDX2, and SATB2, and focally positive for CK7 (Fig. 2). Pelvic washings revealed abundant extracellular mucin, paucicellular with scant reactive mesothelial cells. Peritoneal cytology demonstrated mucicarmine (mucin) staining of the dense ascites. These clinical and pathologic findings were consistent with the diffuse peritoneal adenomucinosis (DPAM) variant of PMP originating from a ruptured LAMN-like ectopic mucinous neoplasm arising from a malignant transformation of an ovarian mature cystic teratoma. This pathological designation is in accordance with the recent 2016 Peritoneal Surface Oncology Group International (PSOGI) consensus for classification and pathologic reporting for PMP and associated appendiceal neoplasia that recommends distinguishing between the histologic grade of the appendiceal primary and the metastatic peritoneal disease that occasionally may be discordant [9].

After multidisciplinary review, completion of CRS and HIPEC was recommended. For the HIPEC portion of the procedure, 40 mg of alkylating agent mitomycin-C (MMC) was employed. The peritoneal cancer index (PCI) was scored and calculated as described by Jacquet and Sugarbaker [10]. Calculated total PCI was 21. A complete macroscopic cytoreduction of all diseases was achieved. Procedures included a greater and lesser omentectomy, omental bursectomy, splenectomy, cholecystectomy, right and left upper quadrant and subhepatic parietal peritonectomies, partial glissonian capsulectomy, appendectomy, removal of small bowel mesenteric and serosal implants, total abdominal hysterectomy, left salpingo-oophorectomy, and pelvic peritonectomy with cytoreduction of mucinous peritoneal disease implants including resection of the upper vagina.

Pathology after complete macroscopic cytoreduction similarly demonstrated abundant pools of paucicellular mucin on the resected abdominopelvic disease-bearing visceral and parietal peritoneal surfaces. Importantly, the appendix was diagnosed as benign on permanent histology. Abundant pools of mucin demonstrated no additional or synchronous malignancy and were confirmatory for the DPAM-variant of PMP. Outpatient genetic counseling revealed no familial and/or genetic predisposition.

The patient’s tumor was sent for molecular profiling and next-generation sequencing. The genetic report showed mutations in ATM at splice site 8268+1G>A, GNAS R201C, and KRAS G12D. The molecular profile was also consistent with tumor-associated microsatellite stability with a tumor mutational burden of 3Muts/Mb. Due to the low-grade nature of the tumor, systemic therapy was not recommended. The patient will undergo a biannual routine examination and cross-sectional imaging. Currently, there is no evidence of disease recurrence 1 year after the procedure.

## Discussion

Mature cystic teratomas (MCTs) otherwise known as dermoid cysts are benign and frequently present as ovarian neoplasms. However, in 0.2–2% of cases, they can undergo malignant transformation, as described in this patient [11]. The majority of these transformations develop in post-menopausal patients between the ages of 50–70 years with squamous cell tumorigenesis, most commonly reported [11]. Our patient presented at age 28 years [12]. MCTs reportedly can also undergo transformation to adenocarcinomas, basal cell carcinomas, melanoma, and neuroendocrine tumors due to the malignant potential of pluripotent cells inherent in these tumors (Table 1).

**Table 1:** Prevalence of malignant transformations of mature cystic teratomas

Histopathological type	Prevalence (% of MT)
Squamous cell carcinoma	80 [13]
Adenocarcinoma	5 [13]
Transitional cell carcinoma	<1 [14]
Malignant melanoma	0.2–0.8 [15]
Thyroid carcinoma	0.1–0.2 [16]
Carcinoid tumor	<0.1 [17]

PMP syndrome typically arises from perforation and/or rupture of an appendiceal mucocele or low-grade appendiceal mucocele neoplasms (LAMN) [18, 19]. Extra-appendiceal etiologies, although rare, can be ovarian in origin [2, 3]. Rare incidences have also been reported in association with neoplastic lesions of other sites including fallopian tube, endocervix, small bowel, colorectum, stomach, gallbladder, lung, breast, pancreas, mucinous cysts of spleen, and urachus [20, 21]. High clinical suspicion and evidence supporting an extra-appendiceal origin requires, firstly and importantly, definitive pathologic confirmation of a normal appendix. Secondly, as published reports describe, IHC staining for SATB2. SATB2 is a protein with restricted expression to glandular cells of the lower gastrointestinal tract and frequent association with appendiceal mucinous neoplasms. Lastly, expression of CK20, CDX2, and CK7 corroborates clinically in our patient with a PMP-derived ruptured appendiceal mucinous-like tumor arising from a secondary origin, a malignant transformed ovarian mature cystic teratoma [1, 3].

The decision to perform cytoreductive surgery is contingent upon disease biology, disease burden, and performance status of the patient. In general, limited abdominopelvic peritoneal metastasis or low peritoneal cancer index (PCI) with a low-grade tumor biology is a clinical presentation most amenable to this treatment option in healthy patients [22]. Expert pathologic review is essential prior to proceeding to surgery. Intraoperatively, PCI is determined by scoring the size and/or confluence of disease and calculating the total score derived from 13 regions of the abdomen and pelvis (score range, 0–3; total PCI range, 0–39) [10]. The principles of surgical cytoreduction of tumor are predicated upon six peritonectomy and visceral resective procedures that remove and/or strip cancer from peritoneal abdominopelvic and visceral surfaces as described by Sugarbaker [23]. Importantly, the pharmacokinetic and clinical efficacy of heated intraperitoneal regional perfusion of chemotherapy or HIPEC, in part, relies upon successful removal of macroscopic disease and drug(s) employed [22]. In our patient’s case, a complete cytoreduction was performed and mitomycin C, a potent alkylating chemotherapeutic agent with a high molecular weight (334 Daltons) conducive to intraperitoneal drug retention was utilized to provide an optimal therapeutic concentration time curve ratio with thermal temperature enhancement and tumor penetrance [24].

The HIPEC procedure is routinely carried out using a closed abdomen technique. After cytoreduction of tumor, inflow, and outflow drainage catheters connected to a perfusion pump are placed within the pelvis and upper abdomen, respectively. The patient’s skin is closed around the catheters with accompanying temperature probes. Regional perfusion of crystalloid containing heated chemotherapy at moderate hyperthermia temperatures (40–44 °C) is circulated and mechanically distributed throughout the abdomen and pelvis for a predetermined period of time in accord with the antineoplastic and chemotherapeutic properties of the agent(s) employed. At the conclusion, drainage catheters and chemotherapeutic perfusate are removed.

Historically, the first patient treated with hyperthermic intraperitoneal chemotherapy was in 1979 for recurrent PMP, having previously undergone CRS. Subsequent phase I and phase II clinical trials in the 1980s demonstrated the effectiveness of intraperitoneal chemotherapy with improved survival in patients largely suffering from ovarian and appendiceal cancer. Currently, CRS and HIPEC are regarded as the standard of care for the treatment of PMP [5]. Recently, efficacy and survival benefit using HIPEC was reported by van Driel et al. when implemented as a treatment strategy during interval cytoreduction for stage III epithelial ovarian cancer in a multicenter, randomized, phase 3 trial [25]. Moreover, this treatment approach has shown particular benefit for abdominal malignancies that include peritoneal mesothelioma, colorectal, and gastric carcinomas [22].

Advanced abdominal malignancies, as in our patient, frequently present with significant tumor burden and upper abdominal, hepatobiliary involvement. There is little debate that suboptimal CRS offers no benefit to the patient. At our institution, cytoreductive surgery frequently includes extensive upper abdominal procedures involving consultant hepatobiliary, gastrointestinal (WBB, AN, HL) along with gynecologic surgical oncologists (SDR, NGR) to optimize the chance for complete macroscopic removal of tumor [8]. As our patient report entails, PMP arising from a malignant transformed ovarian primary mature cystic teratoma is a *rare* “diagnosis of exclusion.” As demonstrated in this current case, it initially masqueraded as an advanced gynecologic malignancy but on subsequent pathologic review resembled the DPAM-variant of PMP arising from a ruptured appendiceal-like neoplasm originating from a MCT. Importantly from a therapeutic standpoint, complete cytoreduction and HIPEC for the DPAM-variant of PMP has a 10-year survival rate of 68% [6]. Importantly, specific experience applying CRS-HIPEC for malignant transformed ovarian teratomas with peritoneal dissemination, although rarely reported, describes potential treatment efficacy with modest disease recurrence and survival [11].

Regardless of origin, PMP has a propensity to recur despite optimal therapy. Similar to traditional appendix-derived PMP, our patient’s tumor harbored non-targetable mutant KRAS-GNAS co-mutations with ATM mutant positivity [26]. Presence of these mutations indeed has a predominant biological impact [27]. To date, current tumor grade pathologic classification systems for PMP (low grade in our patient) have yet shown to directly correlate with modern molecular biomarkers. Advances in molecular characterization of PMP will further enhance our understanding to predict tumor biology and improve identification of targetable, druggable therapeutic biomarkers to prevent disease recurrence [26].

## Conclusion

The present case represents an unusual occurrence and presentation of a rare clinical entity: a PMP syndrome arising in appendiceal-like tissue from a mature cystic teratoma. Discovery of a mature cystic teratoma with malignant transformations coincident with a PMP syndrome should promptly lead to an investigation into the malignancy status of the appendix. During surgery for removal of the mass and/or cytoreduction, the appendix should be removed and submitted for pathologic analysis. Notably, CRS and HIPEC are recommended treatments for appendiceal-derived PMP. However, the question will remain whether “proof of concept” performance of CRS and HIPEC will ultimately contribute to similar durable disease control with PMP-derived from a transformed MCT. Our case provides a clinical clue and suggests that it does. This report adds to the existing evidence supporting this aggressive treatment option in these unique patients. Limitations of this case report include the retrospective nature of this review, duration of follow-up, and requirement for more patients to validate this treatment strategy.

## Data Availability

Data sharing is not applicable to this article as no datasets were generated or analyzed during the current study.
